# Expectancies, Socioeconomic Status, and Self-Rated Health: Use of the Simplified TOMCATS Questionnaire

**DOI:** 10.1007/s12529-012-9221-x

**Published:** 2012-02-01

**Authors:** Magnus Odéen, Hugo Westerlund, Töres Theorell, Constanze Leineweber, Hege R. Eriksen, Holger Ursin

**Affiliations:** 1 Uni Health, Uni Research, Bergen, Norway; 2 Stress Research Institute, Stockholm University, Stockholm, Sweden; 3 Clinic Physical Medicine and Rehabilitation, Kysthospitalet, Vestfold Hospital Trust, Stavern, Norway; 4 Department of Health Promotion and Development, Faculty of Psychology, University of Bergen, Bergen, Norway; 5 Uni helse, Postboks 7810, 5020 Bergen, Norway

**Keywords:** Cognitive Activation Theory of Stress (CATS), Socioeconomic gradient in health, TOMCATS inventory, Coping, SES ladder, Norway, Sweden

## Abstract

**Background:**

Coping has traditionally been measured with inventories containing many items meant to identify specific coping strategies. An alternative is to develop a shorter inventory that focusses on coping expectancies which may determine the extent to which an individual attempts to cope actively.

**Purpose:**

This paper explores the usefulness and validity of a simplified seven-item questionnaire (Theoretically Originated Measure of the Cognitive Activation Theory of Stress, TOMCATS) for response outcome expectancies defined either as positive (“coping”), negative (“hopelessness”), or none (“helplessness”). The definitions are based on the Cognitive Activation Theory of Stress (CATS; Ursin and Eriksen, Psychoneuroendocrinology, 29(5):567–92, 2004). The questionnaire was tested in two different samples. First, the questionnaire was compared with a traditional test of coping and then tested for validity in relation to socioeconomic differences in self-reported health.

**Methods:**

The first study was a comparison of the brief TOMCATS with a short version of the Utrecht Coping List (UCL; Eriksen et al., Scand J Psychol, 38(3):175–82, 1997). Both questionnaires were tested in a population of 1,704 Norwegian municipality workers. The second study was a cross-sectional analysis of TOMCATS, subjective and objective socioeconomic status, and health in a representative sample of the Swedish working population in 2003–2005 (*N* = 11,441).

**Results:**

In the first study, the coping item in the TOMCATS questionnaire showed an expected significant positive correlation with the UCL factors of instrumental mastery-oriented coping and negative correlations with passive and depressive scores. There were also the expected correlations for the helplessness and hopelessness scores, but there was no clear distinction between helplessness and hopelessness in the way they correlated with the UCL. In the second study, the coping item in TOMCATS and the three-item helplessness scores showed clear and monotonous gradients over a subjective socioeconomic status (SES) ladder. Positive response outcome expectancy (“coping”) was related to high subjective SES and no expectancy (“helplessness”) to low subjective SES. In a model including age and sex, TOMCATS scores explained more variance (*r* ^2^ = 0.16) in self-reported health than both subjective (*r* ^2^ = 0.08) and objective SES (*r* ^2^ = 0.02).

**Conclusion:**

The brief TOMCATS questionnaire showed acceptable and significant correlations with a traditional coping questionnaire and is sensitive enough to register systematic differences in response outcome expectancies across the socioeconomic ladder. The results furthermore confirm that psychological and learning factors contribute to the socioeconomic gradient in health.

## Introduction

The traditional way of testing coping is through questionnaires with a large number of questions based on definitions of coping as strategies (e.g. “Ways of Coping”) [1]. Whilst these questionnaires yield differentiated assessments of the coping styles used by the respondents, such long questionnaires are often impractical in epidemiological research and clinical settings. Furthermore, it can be argued that the most important aspect of coping for health outcomes is not *how* a person copes but rather *if* the person tries to cope at all.

In contrast to other theories of coping, the Cognitive Activation Theory of Stress (CATS) [2] stipulates a formal set of definitions for the mechanisms that dampen, eliminate, or reinforce the stress response to a challenging situation. All individuals have acquired such expectancies in relation to stimuli and to the outcome of the responses that are available. The response outcome expectancies are categorised as either positive (coping, expected to lead to a positive outcome), negative (hopelessness), or uncertain (helplessness). Based on CATS, we have developed Theoretically Originated Measure of the Cognitive Activation Theory of Stress (TOMCATS), a brief questionnaire aiming to measure response outcome expectancies.

There have been a number of studies comparing single-item questions with established measures. Good predictive and converging validity has been demonstrated on a number of concepts such as general health [3], burnout [4], patient satisfaction [5], self-esteem [6] and anxiety [7]. With depression, the experience with single-item questions is inconsistent. Asking “are you depressed” to cancer patients worked well compared with a full diagnostic interview in North America [8], but not in Japanese [9] or UK [10] cancer patients. Reme and Eriksen [11] found that a single depression question identified most of the depressive symptoms measured by the Hopkins Symptom Checklist-25 in chronic pain patients. In study 1, we tested the concurrent validity of TOMCATS by comparing it with a traditional test of coping strategies. In study 2, we examined the relations between the questionnaire and socioeconomic differences in health in order to test the validity of TOMCATS.

The presence of substantial socioeconomic differences in health is well established [12] and is often manifested as gradients rather than differences between distinct classes [13]. Explanations offered for socioeconomic differences in health may be classified into two, possibly interacting, categories: structural vs. individual factors. Structural factors are external to the individual, such as the social and societal context, for instance differences in wealth, access to education and physical environment. Individual factors are internal to the individual, such as health behaviours, expectancies, intelligence, or social skills. TOMCATS measures the individually acquired expectancies of being able, or unable, to handle the stressors and challenges of everyday life. In this design, we test whether this brief questionnaire reveals any new perspective on the relations between socioeconomic factors and health.

Our main hypothesis is that differences in socioeconomic status (SES) over the life course lead to differences in reinforcement contingencies, which in turn lead to differences in response outcome expectancies. Furthermore, we hypothesise that individual differences in response outcome expectancies contribute to the socioeconomic differences in health, for instance through differences in health behaviours [14]. In most work on SES and health, objective measures of SES have been used. In this article, we added a scale measuring the individual’s subjective evaluation of his or her place in society. This scale should be more sensitive to the learning history that we believe to be an essential factor for differences in health, particularly for subjective evaluation of health.

## Methods

### Sample and Procedure

#### Study 1

The TOMCATS inventory and a short version of the Utrecht Coping List [15] were presented to a sample of 2,097 Norwegian municipality workers (mean age, 44 years; 81% female). Of these, 1,704 responded to all coping questions and were included in the analysis.

The data were collected in 2008 and 2009 as part of a randomised controlled trial in the process of being published elsewhere. Information about the project was provided through a series of meetings with managers at all levels in the municipalities. The managers provided all employees with information about the study, including an information flyer, informed consent forms and questionnaires. These were then returned to Uni Health in sealed envelopes. The study was approved by the regional research ethics board in western Norway (REK-Vest) and at the Norwegian National Hospital (Rikshospitalet), as well as the Norwegian Social Science Data Services.

#### Study 2

This report is based on the 2008 data wave of the Swedish Longitudinal Occupational Survey of Health (SLOSH), a longitudinal cohort approximately representative of the Swedish working population in 2003–2005 [16, 17]. SLOSH consists of register data and biennial mail-out questionnaires sent to the respondents of the Swedish Work Environment Surveys (SWES) conducted in 2003 and 2005. SWES comprises a stratified random sample of the respondents in the Labour Force Survey from the same years who stated that they were currently working gainfully. A detailed description of the selection process is given elsewhere [17]. There are 18,915 persons in the SLOSH cohort, and 61% answered the survey in 2008, yielding an analytic sample of 11,441 participants.

The analytic sample consisted of 55% women, and the mean age was 49 years (range, 19–70 years, SD = 11.6). Of the participants, 9,588 were employed (85%) and 1,624 were not gainfully employed at the time of the 2008 survey. Fifty-six per cent were married, 88% had at least high school education, and 36% had 2 years of university education or more. Three per cent had unskilled manual jobs, 43% had skilled manual jobs, 23% had non-manual jobs, and 30% had professional or higher management jobs. The median annual income before tax was 298,000 SEK, with a standard deviation of 171,250 SEK. At the time, this was roughly equal to 31,850 € or US $49,140.

The data were collected by Statistics Sweden (SCB) as a pen-and-paper postal survey in two editions: one for respondents in work and one for non-working respondents. All participants received both questionnaires and were asked to fill out the edition that matched their current employment status. Those who worked gainfully 30% or more filled out the worker version and the others filled out the non-worker version. All questionnaires consisted of three parts: Part 1 covers work or current situation as pensioner, unemployed, etc., part 2 covers health, and part 3 health behaviours and social situation outside of work. Both the working group and the non-workers answered all the questions used in this article. On questions regarding job title, non-workers were asked about their previous job. After adding register data, SCB delivered a de-identified data set to the researchers. The study was approved by the regional research ethics board in Stockholm.

#### Instruments

##### Theoretically Originated Measure of the Cognitive Activation Theory of Stress

TOMCATS is a new measure designed to measure the concept of response outcome expectancies as defined in the CATS theory [2, 18]. In study 1, six of the final seven items were used. The inventory consists of three factors that represent the three response outcome expectancy dimensions of CATS: positive expectancy (one item), no expectancy (two items) and negative expectancy (three items). All items were rated on a four-point scale from “not true at all” to “completely true”. The questions are translated to English, but it should be noted that the results in this report are based on the Norwegian and the Swedish versions; the English translation has not been tested.

In study 2, the questions were translated to Swedish by Uni Health and the Stress Research Institute at Stockholm University. Two extra items were added to the version used in study 1, but since only the item “*All my attempts at changing my life are meaningless*” contributed to the helplessness factor, the other item was excluded from the analyses. The final version of the scale consisted of seven items: one for coping and three each for helplessness and hopelessness. Due to a layout error at the printing of the forms, the scale of the last three items was reversed in some of the forms given to the employed respondents. To correct for this, those who responded “completely true” on the coping item, and “somewhat true” or “completely true” on the hopelessness items were excluded since their answers were self-contradictive and probably due to the scale reversal. Using this procedure, 210 out of 11,441 respondents were excluded, i.e. 1.8% of the sample.

##### UCL (Study 1)

The short Norwegian version of the Utrecht Coping List (UCL) [15, 19] consists of 22 items measuring active problem solving, passive avoidance and depressive reaction pattern. Instrumental Mastery-Oriented Coping (IMOC) was used as a measure of positive response outcome expectancy [15]. The IMOC is calculated as the sum of the active problem solving and the inverse of the passive avoidance and depressive reaction pattern. Thus, a person with a high score on active problem solving and low scores on the depressive reaction pattern and passive expectancy would have a very high score on the IMOC factor.

##### Demographic Data (Study2)

Sex and age at inclusion were derived from the ten-digit personal identification number used by Swedish authorities. Education level and income were obtained from register data. Education was classified into five categories: less than high school, high school, <2 years of undergraduate studies, ≥2 years of undergraduate studies and graduate studies. Income was obtained from the 2006 tax report and measured in units of 1,000 SEK and transformed into US dollars and Euros according to the exchange rate on 2 May 2008. It includes all income before taxation, such as salaries, investment profits, interests and public benefits.

##### Occupation Title (Study 2)

This was obtained from the Labour Force Survey in 2003 and 2005, respectively, where the respondents were asked to give their specific work title. The titles were then classified according to the third version of the International Standard Classification of Occupations (ISCO-88). Non-working respondents were asked to report the job they used to have (or the job they had held for the longest period). The occupations were then grouped into five categories: “professionals and higher managers”, “technical, lower management”, “non-manual”, “skilled manual” and “unskilled”. Those in the armed forces (*N* = 26) were excluded since it was difficult to classify them into the five categories in any meaningful way.

##### Health (Study 2)

This was measured by a single question: “How would you rate your general state of health?” Respondents were given five alternatives from “very good” to “very bad”. The scoring of the answers was then reversed and given a score from 1 to 5, with low scores representing poor health. The one question on health has been extensively validated as a valid measure of health in several large studies in Europe and the USA [20–22].

##### Subjective Socioeconomic Status—The SES Ladder (Study 2)

In most work on SES and health, objective measures of SES have been used. In this article, we added a scale measuring the individual’s subjective evaluation of his or her place in society. The MacArthur Scale of Subjective Social Status [23] is a measure designed to capture an individual’s subjective evaluation of her social status relative to society. The respondent is told that the top of the ladder indicates those with the best education, most money, and the best jobs and that the lowest rank has the least amount of money and education, and the worst or no jobs. The respondent then marks a rung in a ladder of ten rungs, and this is translated to a score of 1 to 10. Subjective SES has been found to be associated with both physical and mental health often more strongly than objective measures [23–25].

### Statistics

#### Study 1

Bivariate correlations were calculated with a list-wise deletion so that the sample was identical for all analyses. In order to control for distribution of the data, they were transformed logarithmically, but this did not change any significance level or change the correlations substantially, so the uncorrected data were used. SPSS statistics version 19 was used for the analysis.

#### Study 2

The TOMCATS inventory was tested with a principal components factor analysis with the varimax rotation method. We chose to specify a three-factor solution as the CATS theory clearly specifies three possible conditions of expectancy [2]. Cronbach’s alpha was used to test the internal consistency of the identified factors.

Linear hierarchical multiple regression analyses were used to assess the relationship between coping, SES and health whilst controlling for demographic variables. The correlations between the predictor variables, as well as the variable inflation factor, were examined to control for multicollinearity in the analysis.

Fisher’s protected *t* test [26] was used to control for multiple testing. Under this criterion, significance must be found both at the *r* ^2^ of the regression step and for the beta value of the individual variable to be considered a significant predictor. In order to assess the relative association between the different sets of variables and the outcome variable, the variables were added and removed in different steps. All the response outcome expectancy variables and the income variable were logarithmically transformed to correct for non-normal distribution. SPSS version 19 was used for the analyses.

## Results

### Study 1

The results showed an expected significant positive correlation between the coping item in TOMCATS and the UCL Instrumental Mastery-Oriented Coping Scale. The item correlated positively with UCL active coping and had negative correlations with passive and depressive scores (see Table 1). There were also negative correlations between instrumental mastery-oriented coping and the TOMCATS helplessness and hopelessness scores, and moderate but significant correlations between the TOMCATS helplessness and hopelessness scores and the UCL passive and depressive scores. However, the helplessness and hopelessness factors had similar correlations with passive and depressive reaction pattern; the hopelessness factor was not more highly correlated with the passive than the depressive reaction pattern.

**Table 1:** Correlations between the TOMCATS inventory and the Utrecht Coping List in 1,702 Norwegians

	IMOC	Active	Passive	Depressive
Coping	0.30	0.27	−0.18	−0.22
Helplessness	−0.44	−0.18	0.31	0.45
Hopelessness	−0.47	−0.23	0.34	0.47

All correlations are significant (*p* < 0.001)

*IMOC* Instrumental Mastery-Oriented Coping, *Active* active problem solving, *Passive* passive avoidance, *Depressive* depressive reaction pattern

### Study 2

#### The TOMCATS Inventory

The Kaiser–Mayer–Olkin value was high (0.80), and Bartlett’s test of sphericity was significant (*p* < 0.001). Three distinct factors were identified: hopelessness (initial eigenvalue, 3.50), helplessness (initial eigenvalue, 1.63) and coping (initial eigenvalue, 0.91; see Table 2). Item 5 was discarded since it decreased the Cronbach’s alpha of the helplessness (from 0.90 to 0.82) and did not increase the Cronbach’s alpha of the hopelessness factor.

**Table 2:** Rotated factor loadings in a principal component analysis with a varimax rotation on the TOMCATS inventory in 10,959 Swedes. Underlining indicates which items belong to which factors

		Helplessness	Hopelessness	Coping
1	I can solve most difficult situations with a good result	−0.13	−0.11	0.98
2	I really don’t have any control over the most important issues in my life	0.70	0.09	−0.14
3	I wish I could change my life, but it’s not possible	0.84	0.08	−0.09
4	All my attempts at changing my life are meaningless	0.85	0.15	−0.05
5	It is better that I don’t try to solve my own problems, so that I don’t make problems for myself and others	0.71	0.26	0.03
6	It’s better that others try to solve my problems than for me to mess things up and make them worse	0.13	0.88	−0.07
7	I would have been better off if I didn’t try so hard to solve my problems	0.18	0.90	−0.06
8	All my attempts at making things better just makes them worse	0.17	0.90	−0.06
Rotation sum of squared loadings		2.53	2.51	1.00
% of variance		31.63	31.37	12.51
Cronbach’s alpha		0.895	0.796	N/A (only 1 item)

#### CATS and SES

**Fig. 1:**
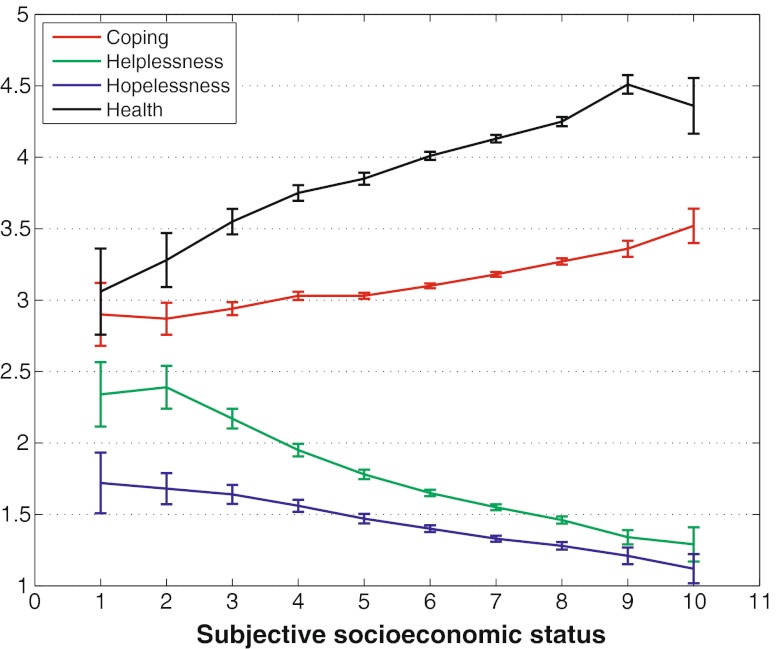
Gradients of health, coping and depression in the Swedish population. The scale of the health question and the response outcome expectancies is represented on the *Y*-axis. Health has a range from 1 to 5 and response outcome expectancies from 1 to 4

All expectancy variables (coping, helplessness and hopelessness) showed clear (*p* < 0.001) and monotonous gradients over the subjective SES ladder (see Fig. 1). As expected, coping was positively associated with social status, whilst hopelessness and helplessness showed a clear negative association with subjective SES scores (see Table 3 for means and standard deviations for all ten steps of the SES ladder).

**Table 3:** Response outcome expectancies, health and subjective social status (*N* = 10,776)

Ladder position	*N*	Coping	Helplessness	Hopelessness	Health
		Mean (SD)			
1 Lowest	57	2.90 (0.85)	2.34 (0.87)	1.72 (0.82)	3.06 (1.16)
2	133	2.87 (0.66)	2.39 (0.88)	1.68 (0.64)	3.28 (1.11)
3	409	2.94 (0.47)	2.17 (0.71)	1.64 (0.68)	3.55 (0.92)
4	936	3.03 (0.46)	1.95 (0.69)	1.56 (0.66)	3.75 (0.85)
5	1,497	3.03 (0.42)	1.78 (0.66)	1.47 (0.66)	3.85 (0.83)
6	2,733	3.10 (0.45)	1.65 (0.60)	1.40 (0.63)	4.01 (0.76)
7	3,093	3.18 (0.46)	1.55 (0.57)	1.33 (0.60)	4.13 (0.75)
8	1,935	3.27 (0.50)	1.46 (0.56)	1.28 (0.60)	4.25 (0.73)
9	362	3.36 (0.55)	1.34 (0.48)	1.21 (0.57)	4.51 (0.63)
10 Highest	75	3.52 (0.53)	1.29 (0.53)	1.12 (0.45)	4.36 (0.86)
*p* for trend^a^	<0.001	<0.001	<0.001	<0.001	

The range for all TOMCATS variables is 1-4. The range of the Health variable is 1-5

^a^Response outcome expectancy variables were logarithmically transformed in calculating the values

Bivariate correlations between the variables were examined to control for multicollinearity (see Table 4). The variance inflation factor was below 2.0 for all predictors in all regression analyses. In a multiple regression analysis, response expectancies had significant associations with age, gender and subjective or objective social status (see Table 5). The results were very similar for all the response outcome expectancies, with subjective social status as the best predictor.

**Table 4:** Bivariate correlation matrix for variables included in the analyses of study 2 (*N* = 10,445)

	General health	Age	Gender	Subjective SES	Income	Job	Education	Coping	Helplessness	Hopelessness
General health	1	−0.06	0.02	0.27	0.06	0.09	0.09	0.21	−0.37	−0.21
Age	−0.06	1	−0.05	0.06	0.19	0.03	−0.13	−0.04	−0.04	−0.02
Gender	0.02	−0.05	1	−0.06	−0.22	−0.01	0.13	−0.03	−0.01	−0.01
Subjective SES	0.27	0.06	−0.06	1	0.25	0.36	0.26	0.20	−0.30	−0.19
Income	0.06	0.19	−0.22	0.25	1	0.32	0.14	0.06	−0.05	−0.05
Job	0.09	0.03	−0.01	0.36	0.32	1	0.62	0.08	−0.05	−0.10
Education	0.09	−0.13	0.13	0.26	0.14	0.62	1	0.06	−0.02	−0.08
Coping	0.21	−0.04	−0.03	0.20	0.06	0.08	0.06	1	−0.23	−0.28
Helplessness	−0.37	−0.04	−0.01	−0.30	−0.05	−0.05	−0.02	−0.23	1	0.39
Hopelessness	−0.21	−0.02	−0.01	−0.19	−0.05	−0.10	−0.08	−0.28	0.39	1

**Table 5:** Hierarchical linear multiple regression analyses with the three TOMCATS factors (coping, hopelessness, and helplessness) as dependent variables and subjective and objective SES (income, work education) as independent variables, controlled for age and sex and including *F* values and degrees of freedom for regression and residual

	Age, *β*	Sex, *β*	SES ladder, *β*	Education, *β*	Income, *β*	Work, *β*	Total		
							*F* value	*df* (reg/res)	Adjusted *R* ^2^ (*p* value)
Coping (*N* = 10,736)									
Step 1	−0.041**	−0.036**					15.53	2/10,733	0.003 (<0.001)
Step 2	−0.052**	−0.025*	0.191**				145.44	3/10,732	0.039 (<0.001)
Step 3	−0.049**	−0.028*		0.007	0.041**	0.061**	22.40	5/10,730	0.010 (<0.001)
Step 4	−0.56**	−0.021*	0.185**	−0.011	0.015	0.015	73.42	6/10,729	0.039 (<0.001)
Helplessness (*N* = 10,743)									
Step 1	−0.042**	−0.011					9.90	2/10,740	0.002 (<0.001)
Step 2	−0.026	−0.028*	−0.293**				342.75	3/10,739	0.087 (<0.001)
Step 3	−0.034*	−0.019		0.007	−0.032*	−0.014*	9.70	5/10,737	0.004 (<0.001)
Step 4	−0.023*	−0.032*	−0.322**	0.039*	0.014*	0.043*	182.35	6/10,736	0.092 (<0.001)
Hopelessness (*N* = 10,558)									
Step 1	−0.020*	−0.013					2.91	2/10,555	0.000 (0.054)
Step 2	−0.010	−0.024*	−0.194**				138.31	3/10,554	0.038 (<0.001)
Step 3	−0.017	−0.015		−0.031	−0.025*	−0.076**	26.27	5/10,552	0.012 (<0.001)
Step 4	−0.011**	−0.022*	−0.179**	−0.014	−0.000	−0.031*	9.04	6/10,551	0.039 (<0.001)

Step 1: Age and gender; Step 2: Age, gender, subjective SES; Step 3: Age, gender, objective SES; Step 4: Age, gender, and subjective and objective SES

*Beta* standardised linear regression coefficient, *SE* standard error of the mean

*p < 0.05, **p < 0.001 (predictor value)

#### The SES Health Gradient

The health variable also showed clear gradients (*p* < 0.001) over the subjective SES scale, consistent with expectations (see Table 3). After controlling for age and gender, SES was a weak but significant predictor of health, both when measured as a subjective rating (*r* ^2^ = 0.078) or by objective measures of income, education and job (*r* ^2^ = 0.015; see Table 6).

**Table 6:** Linear hierarchical multiple regression with perceived health as outcome, subjective and objective socioeconomic status (income, work and education), and the factors of the TOMCATS inventory as independent variables, including *F* values and degrees of freedom for regression and residual (*N* = 10,445)

		Step 1	Step 2	Step 3	Step 4	Step 5	Step 6
*β* (SE)	Age	−0.057 (0.010)**	−0.072 (0.009)**	−0.063 (0.010)**	−0.068 (0.009)**	−0.070 (0.009)**	−0.075 (0.009)**
	Gender	0.014 (0.010)	0.030 (0.009)*	0.020 (0.010)	0.013 (0.009)	0.016 (0.009)	0.022 (0.009)*
	SES ladder		0.274 (0.09)**				0.161 (0.009)**
	Education			0.038 (0.13)*			
	Income			0.014 (0.11)**			
	Work			0.012 (0.13)**			
	Coping				0.121 (0.009)**	0.116 (0.009)**	0.101 (0.009)**
	Helplessness				−0.330 (0.010)**	−0.331 (0.010)**	−0.291 (0.010)**
	Hopelessness				−0.044 (0.010)**	−0.038 (0.010)**	−0.034 (0.010)**
Total	*F*	18.31	294.62	30.84	403.83	262.92	394.93
	*df* (reg/res)	2/10,442	3/10,441	5/10,439	5/10,439	8/10,436	6/10,438
	Adjusted *R* ^2^ (*p*)	0.003 (<0.001)	0.078 (<0.001)	0.014 (<0.001)	0.162 (<0.001)	0.167 (<0.001)	0.185 (<0.001)

Step 1: Age, gender; Step 2: Age, gender, subjective SES; Step 3: Age, gender, objective SES; Step 4: Age, gender, response expectancies; Step 5: Age, gender, objective SES, response expectancies; Step 6: Age, gender, subjective SES, response expectancies

*Beta* standardised linear regression coefficient, *SE* standard error of the mean

*p < 0.05, **p < 0.001 (predictor value)

#### Health

In a multiple regression analysis of the determinants of perceived health, response outcome expectancies were the best predictors for health. The model containing only the response outcome expectancies explained almost as much variance as the models that included subjective or objective SES. In the full model, the most important variables were helplessness (*β* = −0.29), subjective SES (*β* = 0.15) and coping (*β* = −0.10; see Table 6).

## Discussion

The results from study 1 showed the expected significant correlations between the coping item in TOMCATS and the Utrecht Coping List (UCL): positive with overall instrumental mastery-oriented coping as well as with the active coping subscale and negative with passive coping and depressive scores. There were also negative correlations between instrumental mastery-oriented coping and the TOMCATS helplessness and hopelessness scores, and moderate but significant correlations between the TOMCATS helplessness and hopelessness scores and the UCL passive and depressive scores. However, the helplessness and hopelessness factors had similar correlations with passive and depressive reaction patterns, and contrary to expectation, the hopelessness factor was not more strongly correlated with the passive than the depressive reaction pattern.

Previous validation studies with coping instruments have shown a wide range of results. Correlations in the order of 0.78 were found when measuring the same coping concept (general self-efficacy, GSE) with two different general self-esteem scales [27]. When measuring against similar but not identical concepts, correlations between GSE and the positive emotions factor of the “Positive and Negative Affect Scale” were about 0.40. The correlation with the less general concept of “health locus of control” was 0.23, a low but significant correlation [28].

The UCL was chosen as a validation instrument because the instrument specifies a structure similar to the TOMCATS inventory by separating a passive avoidance strategy from a depressive reaction pattern. However, there are important distinctions as the UCL measures strategies and TOMCATS measures expectations, so we did not expect to see very high correlations between the factors. The fact that TOMCATS showed meaningful and moderate correlations indicates a relation to the coping strategies without measuring exactly the same phenomena. However, the correlations were somewhat low (between 0.27 and 0.47), which indicates that the TOMCATS factors are less closely related to the UCL than expected, but the results were generally in line with previous studies of closely related but different concepts.

Study 2 showed a strong association between the subjective expectation of coping, SES and self-rated general health. This supports the assumption that individual-learned expectancies matter for socioeconomic health differences [14, 29]. As expected, low social status was also associated with individual experiences of failure to cope with the challenges of life (hopelessness) and the expectancy that there is no predictable relationship between what the individual does and what happens to him or her (helplessness). The gradient for helplessness appears even more pronounced than for hopelessness. This may be because the small number of individuals reporting a high degree of hopelessness creates a floor effect. Furthermore, the moderate amount of explained variance in our models indicates that there is reason for some caution when interpreting the importance of the results.

The two studies indicate the usefulness of a very brief questionnaire testing general response outcome expectancies. Important relations are revealed without the use of long and tedious forms. In a previous report, Odéen et al. [30] analysed two questionnaires: one based on the UCL [15] which is a development of the Lazarus Ways of Coping scales [1] and one based on the Bandura self-efficacy concept [31], the General Perceived Self-Efficacy Scale [32]. None of the questionnaires were able to predict return to work in patients in a rehabilitation clinic. There were also difficulties with the theoretical bases as the questionnaires identify general trends rather than specific strategies. Given this lack of precision of the two instruments and the theoretical problems with them, the authors felt that a moderate degree of caution is warranted when inferring from results from these questionnaires to CATS or self-efficacy theory. The general overarching brief questions used in TOMCATS may be a better way to catch general trends and attitudes.

Our data support the individual explanations of socioeconomic differences in health as coping outcome expectancy is more strongly associated with self-rated health than both objective and subjective measures of socioeconomic status. However, structural factors in the social environment influence the learning history of an individual through differences in reinforcement contingencies. Those who grow up in high social strata have more resources available, and the chances of experiencing positive outcomes of coping attempts are probably higher. There is evidence that a low socioeconomic status has negative effects on health from early in life, and there may be “vicious circles” where adverse circumstances contribute to the development of expectancies of no or negative response outcomes of attempted coping [14]. This in turn inhibits motivation to engage in behaviours that could lead to better health [14] and could also mean that the individual is more likely to remain in an unfavourable social position.

If outcome expectancies can explain differences in health, a systematic effort to change the response outcome expectancies early in life could potentially be of great long-term benefit for individuals and reduce the social inequalities in health. It may be this very learning process that determines later behaviour, later optimism and later motivation to take care of one’s own health. A positive response outcome expectancy improves the chances that the individual will choose positive health behaviours. Further confirmation of the theoretical position would be to show that interventions aiming at improving coping skills and expectancies attached to coping strategies improve the health status of individuals. There is a possibility that the relationship between coping and subjective and objective socioeconomic status is reciprocal, in other words that coping is important in key behaviours that may advance or impede social mobility, such as children’s perceived vocational outcomes [33], educational perseverance and performance [34], and job satisfaction and performance [35]. The associations between health, SES and coping suggest a common underlying factor, such as a tendency to view the world in an optimistic or pessimistic way. This may be a crucial element in the many cognitive interventions available to improve both subjective health and loss of function and working capacity.

The main strength of the two studies in this paper is that they are based on large, representative population samples. Mechanisms underlying social gradients could vary between countries and replications in other population samples would strengthen the evidence. It should be noted that in the first study, the sample was fairly homogenous; 80% were women and all were public sector employees. The SLOSH sample, however, was larger and representative of the national working population. Also, low test–retest reliability has been reported for a single global question on health [36], and this tendency was stronger in subjects with low SES.

At this point in time, TOMCATS has been used solely as an explorative tool in epidemiological research. For the instrument to be used for other purposes, such as screening for low coping, clinical use, or as an indicator of effect of interventions, more validation research is needed. Especially, the test–retest reliability of the scale, as well as the sensitivity to change (the smallest detectable change as well as the minimal important change), needs to be established and reported. The CATS theory [2] makes clear predictions of coping as a result of learned expectancies. In order to be a valid instrument based on this theory, TOMCATS must show stability in periods where no or minimal learning of new expectancies takes place, as well as sensitivity to acquisition of new expectancies. This would be a natural next step in the development of the TOMCATS inventory.

The major limitation of both studies in this article is that they are cross-sectional. In order to fully study the associations between environmental factors, coping expectancy and health, life course data would be needed. In addition, the use of objectively assessed, prospective health outcomes would further strengthen the evidence.

## Conclusions

The brief TOMCATS questionnaire showed acceptable and significant correlations with a traditional coping questionnaire and is sensitive enough to register systematic differences in response outcome expectancies across the socioeconomic ladder. The results furthermore confirm that psychological and learning factors contribute to the socioeconomic gradient in health.
